# Inhibition of Dendritic Cell Activation and Modulation of T Cell Polarization by the Platelet Secretome

**DOI:** 10.3389/fimmu.2021.631285

**Published:** 2021-02-25

**Authors:** Anno Saris, Juulke Steuten, David P. Schrijver, Gijs van Schijndel, Jaap Jan Zwaginga, S. Marieke van Ham, Anja ten Brinke

**Affiliations:** ^1^ Department of Immunopathology, Sanquin Research and Landsteiner Laboratory, Amsterdam University Medical Center, University of Amsterdam, Amsterdam, Netherlands; ^2^ Department of Infectious Disease, Leiden University Medical Center, Leiden, Netherlands; ^3^ Center for Clinical Transfusion Research, Sanquin Research, Leiden, Netherlands; ^4^ Department of Hematology, Leiden University Medical Center, Leiden, Netherlands; ^5^ Swammerdam Institute for Life Sciences, University of Amsterdam, Amsterdam, Netherlands

**Keywords:** platelet immunomodulation, transfusion-related immune modulation, platelet releasate, monocyte-derived dendritic cells, primary dendritic cell, T cell priming

## Abstract

Platelet transfusions are a frequently administered therapy for especially hemato-oncological patients with thrombocytopenia. Next to their primary function in hemostasis, currently there is increased attention for the capacity of platelets to affect the function of various cells of the immune system. Here, we investigate the capacity of platelets to immuno-modulate monocyte-derived dendritic cells (moDC) as well as primary dendritic cells and effects on subsequent T cell responses. Platelets significantly inhibited pro-inflammatory (IL-12, IL-6, TNFα) and increased anti-inflammatory (IL-10) cytokine production of moDCs primed with toll-like receptor (TLR)-dependent and TLR-independent stimuli. Transwell assays and ultracentrifugation revealed that a soluble factor secreted by platelets, but not microvesicles, inhibited DC activation. Interestingly, platelet-derived soluble mediators also inhibited cytokine production by human *ex vivo* stimulated myeloid CD1c+ conventional DC2. Moreover, platelets and platelet-derived soluble mediators inhibited T cell priming and T helper differentiation toward an IFN*γ*+ Th1 phenotype by moDCs. Overall, these results show that platelets are able to inhibit the pro-inflammatory properties of DCs, and may even induce an anti-inflammatory DC phenotype, with decreased T cell priming capacity by the DC. The results of this study provide more insight in the potential role of platelets in immune modulation, especially in the context of platelet transfusions.

## Introduction

Up to three million platelet transfusions are administered every year in Europe, and the majority (up to 65%) of these transfusions are administered to hemato-oncological patients (1–7). Prophylactic administration of platelet transfusions in patients not at direct risk of bleeding is currently subject of debate (6–9). Next to the fact that platelet transfusions are costly and involve complicated logistics, they are associated with adverse reactions like alloimmunization, febrile non-hemolytic transfusion reactions (FNHTR), transfusion related acute lung injury (TRALI), as well as the still somewhat elusive transfusion related immune modulation (TRIM) (8, 10–13). TRIM associates with increased risk of infections, decreased graft rejection and recurrence of tumor growth in patients receiving transfusions (14–16). Even though a number of studies have investigated TRIM, the underlying mechanisms remain largely unresolved (17–20).

Platelets are anucleate megakaryocyte-derived cell fragments packed with secretory granules containing many hemostatic and angiogenic factors, but also a collection of mediators that affect the immune system, ranging from chemokines (e.g. chemokine ligand 5 (CCL5)) to microbicidal molecules (e.g. thrombocidin-1 and 2) (21, 22). Platelets also express many molecules such as toll-like receptors (TLRs), integrins and costimulatory molecules through which they can sense pathogen-associated molecular patterns and interact with leukocytes (23–26). Moreover, platelets are the main source of (s)CD40L in plasma, and in CD40L deficient mice transfer of wildtype platelets increased virus-specific IgG titers (27, 28). In contrast, platelet derived active transforming growth factor β (TGF-β), together with lactate, was found to inhibit T cell anti-tumor functions (29). Due to increased understanding of the potential immune functions of platelets, the question arises to what extent transfused platelets will affect the recipient’s immune system (12, 17, 20, 30, 31), which would be especially relevant for hematological patients that are frequently strongly immune-compromised (7).

Dendritic cells (DCs) are instrumental for the initiation and skewing of the adaptive immune response. DCs are equipped with many pattern recognition receptors (PRRs) that recognize various pathogen- and danger- associated molecular patterns (PAMP/DAMP). Upon encounter of pathogenic antigens, DCs can engulf and process these antigens and present antigenic peptides in the context of MHC molecules to T cells. The upregulation of costimulatory molecules and secretion of cytokines is directed by the signaling pathways of the engaged PRR(s), whereby DCs prime and skew the antigen-specific T cells toward a particular immune response (32). Due to the central role of DCs in the adaptive immune response it is important to study the potential immune modulatory effect of platelets on DC effector functions. Previous studies describe variable effects of platelets on DC effector function depending mostly on the presence or absence of an additional activating stimulus (33–43). Although some studies have shown that platelets may reduce DC activation, lipopolysaccharide (LPS) was almost exclusively used for DC stimulation (33, 34, 36–38). Therefore, questions remain on the generalizability and consistency of these effects and more importantly what are the consequences on T cell activation and priming.

In the present study we aimed to advance our understanding of platelet-mediated inhibition of human DCs and how this affects subsequent T cell responses. Our studies revealed that upon activation platelets release soluble factors (platelet releasate) that affect both moDC and primary myeloid CD1c+ conventional DC2 (cDC2) cytokine production induced by a range of TLR-dependent and TLR-independent stimuli with minimal effects on co-stimulatory marker expression. Furthermore, moDCs stimulated in presence of platelets or platelet releasate show less ability to induce T cell proliferation and have decreased capacity to skew naïve T cells toward a Th1 IFN*γ* ^+^ phenotype. Overall, we show here that mediators derived from activated platelets directly impair DC effector functions, which may contribute to the occurrence of TRIM upon transfusion of platelets.

## Materials and Methods

### Human Blood Samples

Leukapheresis products or buffy coats obtained from anonymized Sanquin blood donors were used to isolate monocytes and T cells after written informed consent by donors. Citrated whole blood was obtained to isolate fresh platelets and platelet releasate. All procedures were approved by the Sanquin Ethical Advisory Board and are in accordance with the declaration of Helsinki and Dutch regulations.

### Isolation of Human Monocytes and Differentiation to Immature moDC

Monocytes were isolated using the Elutra Cell Separation System (Gambro, Lakewood, CO, USA) from fresh leukapheresis material or from PBMCs isolated from buffy coats using CD14+ magnetic cell separation (Miltenyi Biotech, Leiden, Netherlands) and subsequently frozen until further use as previously described (13, 44). Purity of monocytes was >90% as determined using flow cytometry with APC labeled anti-CD14 (clone MϕP9, BD biosciences). Upon initiation of culture, monocytes were thawed and differentiated into immature dendritic cells (DCs) as previously described (13, 44).

### Platelet Isolation and Production and Ultracentrifugation of Platelet Releasate

Platelets were isolated from citrated whole blood by centrifugation at 200g for 10 min and supernatant platelet rich plasma was washed (5 min, 1650g) 1:2 with sequestrine buffer (17.5 mM Na2HPO4, 8.9 mM Na2EDTA, 154 mM NaCl, pH 6.9, containing 0.1% [wt/vol] bovine serum albumin, all obtained from Merck Millipore, Amsterdam, Netherlands). After washing, platelets were resuspended in GMP DC serum-free medium (Cellgenix, Freiburg, Germany) containing 100 U/ml penicillin and 100 U/ml streptomycin (Gibco). To collect platelet releasate, platelets were incubated 30 min with 250 µM thrombin receptor-activating peptide 6 amide (TRAP-6) (Bachem, Bubendorf, Switzerland) after which platelets were removed by centrifugation: first 5 min at 1,650 g and subsequently 10 min at 4,000g. If indicated, platelet releasate was hereafter centrifuged to remove microparticles at 100,000g for 1 h in Optima L-100 XP Ultracentrifuge (Beckman Coulter, IN, United States). Experiments that directly compared whole platelets and releasate used releasate and whole platelets from the same donor. Otherwise, releasate was pooled from four or five whole blood donors, aliquoted and stored at −20°C until further use.

### Stimulation of DCs

Immature moDCs were stimulated in the absence or presence of platelets or platelet releasate with a platelet:DC ratio ranging from 80:1 to 1:1 (or equivalent amount of releasate) in GMP DC serum-free medium (Cellgenix, Freiburg, Germany) containing 100 U/ml penicillin and 100 U/ml streptomycin (Gibco). When indicated, DCs were stimulated using 2.5 µg/ml monophosporyl lipid A (MPLA) (Sigma Aldrich, Zwijndrecht, Netherlands), 5 µg/ml pam3CysSerLys4 (PAM3CSK4) (Invivogen, San Diego, CA, USA), 2.5 µg/ml resiquimod (R848) (Alexis Biochemical, Paris, France) or 0.25 µg/ml flagellin *bacillus subtilis* (Invivogen) in combination with 1,000 U/ml IFNγ (Boehringer Ingelheim, Alkmaar, Netherlands) or a cytokine maturation cocktail consisting of 10 ng/ml TNFα, 10 ng/ml IL-1β (both Cellgenix, Freiburg, Germany), and 1 µg/ml prostaglandin E2 (PGE_2_) (Sigma Aldrich, Zwijndrecht, Netherlands). When indicated, releasate or recombinant human TGF-β (R&D Systems, Minneapolis, MN, United States) was pre-incubated with blocking antibody against TGF-β (Clone 1D11, 10 µg/ml, BioXCell, NH, United States) for 30’ at 37°C, 5% CO_2_. For transwell experiments, moDCs were stimulated with MPLA/IFN*γ* in a 96-well transwell system (Corning, pore size ≤0.3 µM) in absence or presence of TRAP-6 activated platelets (platelet:DC ratio 40:1) that were either below the transwell (i.e. in the same compartment as the DCs) or separated by the transwell. After 24 or 48 h at 37°C, 5% CO_2_ culture supernatant was harvested and frozen at −20°C until cytokine measurements.

### Cytokine Measurements

The levels of IL-6 and IL-10 in culture supernatants were measured using compact PeliKine Cytokine ELISA kits (Sanquin Reagents, Amsterdam, Netherlands); the level of TNF-α was determined with Human TNF-α ELISA set (Diaclone, Besançon, France). To determine production of IL-12p40, a combination of two anti-IL-12p40 antibodies was used (coat: clone C11.79 and detection: clone C8.6, Sanquin Reagents, Amsterdam, Netherlands) and recombinant IL-12p40 was used as calibration. The level of human TGF-β1 was measured using DuoSet ELISA kit (R&D Systems, Minneapolis, MN, United States) either after or without chemical activation of the samples according to manufacturer’s protocol. Absorbance was measured at 540nm with SynergyTM 2 (BioTek, Winooski, VT, USA).

### Phenotyping DCs

After 48 h of stimulation, DCs were harvested with 0.4% trisodium citrate (Merck Millipore, Amsterdam, Netherlands) in PBS supplemented with 4 mg/ml human albumin (Albuman, Sanquin Reagents, Amsterdam, Netherlands). DCs were stained 30 min at room temperature with LIVE/DEAD Fixable near-IR Dead Cell Stain Kit (Thermo Fisher Scientific) and subsequently with either PE-labeled anti-HLA-DR (Clone G46-6) or with FITC-labeled anti-CD80 (Clone L307.4), PE-labeled anti-CD40 (Clone 5C3), APC-labeled anti-CD83 (Clone HB15e) and BV421-labeled anti-CD86 (Clone 2331) (all from BD Biosciences) in the presence of 3 mg/ml human γ-globulin (Nanogam, Sanquin, Amsterdam, Netherlands). Samples were measured with BD FACSCanto II and analyzed using Flowjo VX (Beckton, Dickinson & Company, Ashland, OR, United States). Gating strategy involved selection of DCs on FSC/SSC, single cells and viable cells.

### Isolation of Human T Cells

Total human lymphocytes were isolated using the Elutra Cell Separation System (Gambro, Lakewood, CO, USA) from fresh leukapheresis material and frozen until further use. Naïve human CD4+ T cells were isolated from buffy coats. First, the PBMC fraction was isolated by density gradient centrifugation (Lymphoprep, Axis-shield, Alere Technologies AS, Oslo, Norway). From PBMC fraction, CD4+ cells were isolated by negative selection using magnetic separation followed by negative selection of CD45RA population (both Miltenyi Biotech, Leiden, Netherlands). Purity of isolated naïve CD4+ T cells ≥95% as determined by staining with FITC-labeled anti-CD45RA (Clone HI100, eBioscience) and BV421-labeled anti-CD45RO (Clone UCHL1, BD Bioscience).

### Isolation and Stimulation of Primary Blood DCs

Primary blood DCs were isolated from buffy coats as previously described (45). In short, PBMCs were enriched for blood DCs by elutriation with JE-5.0 elutriator (Beckman Coulter, Brea, CA, USA), followed by sorting on FACSAria IIIu or III cell sorter (BD Bioscience, San Jose, CA, USA). For sorting, the cells were stained with APC-labeled anti-CD3 (clone HIT3a), anti-CD14 (clone MϕP9), anti-CD19 (clone SJ25-C1) (all BD Biosciences), PE-Cy7-labeled anti-CD1c (clone L161, Biolegend) and FITC-labeled 6-sulfo LacNAc (Slan)-specific antibody (clone M-DC8, Miltenyi Biotech) and LIVE/DEAD fixable Near-IR Dead Cell Stain Kit (Invitrogen). Different DC subtypes were gated as follows: Slan+ non-classical monocytes as CD3^-^CD14^-^CD19^-^M-DC8^+^ and myeloid cDC2 as CD3^-^CD14^-^CD19^-^CD1c^+^ (see **Supplementary Figure S3** for gating strategy). Purity after sort was >90% as tested by flow cytometry. Primary DCs were rested overnight in GMP DC serum-free medium (Cellgenix) containing 100 U/ml penicillin and 100 U/ml streptomycin (Gibco) at 37°C and 5% CO_2_. After overnight rest, DCs were stimulated with R848 (Alexis Biochemical, Paris, France) at a concentration of 5 µg/ml in absence or presence of platelets or platelet releasate in a 50:1 platelet:DC ratio.

### T Cell Proliferation Assay

After thawing and 1 h resting total T cells or naïve CD4+ CD45RA+ T cells were washed twice with PBS and incubated with 5µM CFSE (Invitrogen) for 20 min at room temperature. After washing, T cells were resuspended in IMDM+5%HS medium and incubated 10:1 (T:DC ratio) with autologous (TT-loaded) or allogeneic DCs. If indicated, DCs were first incubated 1 h at 37°C with 5 µg/ml tetanus toxoid (AJVaccines, Copenhagen, Denmark) before stimulation with MPLA/IFN*γ*. After maturation with MPLA/IFN*γ* or IL-1β/TNFα/PGE_2_, moDCs were washed twice with IMDM + 5%HS medium to remove potential remaining platelets or platelet-derived factors. After 8 days, total T cells were harvested, labeled with LIVE/DEAD Fixable near-IR Dead Cell Stain Kit (Thermo Fisher Scientific) and subsequently with PE-labeled anti-CD4 (Clone SK3) and APC-labeled anti-CD8 (Clone SK1) (both from BD biosciences) in FACS buffer (PBS + 0.5% BSA + 0.02% Azide). After 9-10 days, naïve T cells were stimulated with PMA (100 ng/ml) and ionomycin (1 µg/ml) in presence of Brefeldin A (10 µg/ml) (all from Sigma Aldrich) for 5 h. T cells were harvested and washed, labeled with LIVE/DEAD Fixable near-IR Dead Cell Stain Kit (Thermo Fisher Scientific) at RT and subsequently with BUV395-labeled anti-CD4 (clone SK3, BD Bioscience). Hereafter, cells were fixed for 15 min with 4% paraformaldehyde (Merck, Darmstadt, Germany) after which cells were stained with PECy7-labeled anti-IFN*γ* (Clone B27, BD Bioscience), APC-labeled anti-TNFα (clone Mab11, Biolegend) in Brilliant Stain buffer (BD Bioscience) in permeabilization buffer containing 0.5% Saponin, 1%BSA, and 0.02% azide. Cells were washed and measured on Canto II (total T cells) or LSR Fortessa (both from BD Bioscience). Data were analyzed in Flowjo VX (Beckton, Dickinson & Company, Ashland, OR, United States), gating involved selection of lymphocytes based on FSC/SSC, single cells, live CD4+ cells, proliferation dye dilution and positivity for specific cytokines.

### Statistical Analysis

Graphical presentation and statistical analyses were performed using GraphPad prism v8.02 (GraphPad Software Inc, La Jolla, California, USA). Paired t-tests, repeated measures one-way ANOVA and two-way ANOVA with Tukey post test were used to determine statistical differences in DC and T cell cytokine production, DC co-stimulatory marker expression and viability, and T cell proliferation. *p ≤ 0.05, **p ≤ 0.01, ***p ≤ 0.001.

### Data Sharing Statement

For original data, please contact a.tenbrinke@sanquin.nl

## Results

### Platelets Decrease moDC Pro-Inflammatory Cytokine Production Upon DC Activation With Different Stimuli

**Figure 1:**
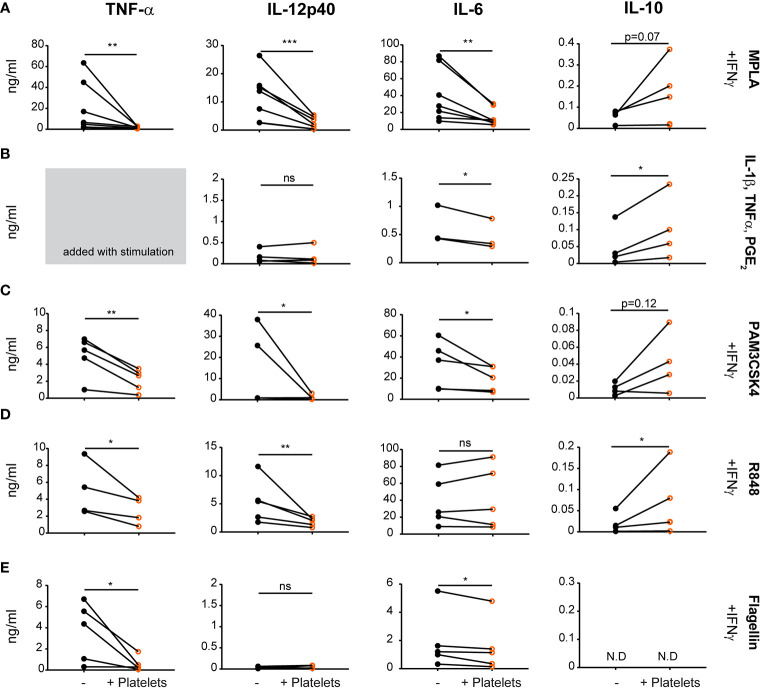
Platelets decrease monocyte-derived dendritic cells (moDC) pro-inflammatory cytokine production upon activation with different stimuli. moDCs were stimulated with **(A)** monophosporyl lipid A (MPLA), **(B)** a cytokine cocktail containing IL-1β, TNFα and prostaglandin E2 (PGE_2_), **(C)** pam3CysSerLys4 (PAM3CSK), **(D)** R848, or **(E)** Flagellin **(A, C–E)** in the presence of IFN*γ*  **(A–E)** in absence or presence of platelets (platelet:DC ratio 40:1). After 48 h, supernatant was harvested and production of TNF-α, IL-12p40, IL-6, and IL-10 was determined by ELISA. Differences in cytokine production (mean ± SEM) were determined by paired t-tests, **(A, C–E)** *n*=5 donors; five independent experiments **(B)** *n*=4 donors; two independent experiments. **(A–E)** IL-6 panel B and IL-10 panel E fewer replicates due to values below detection limit. N.D. not detectable. *p ≤ 0.05, **p ≤ 0.01, ***p ≤ 0.001.

To investigate if and to what extent platelets affect human DC function, monocyte-derived DCs (moDCs) were stimulated with several stimuli to mimic various types of physiological activation, including MPLA (TLR4; **Figure 1A**), PAM3CSK4 (TLR1/2; **Figure 1C**), R848 (TLR7/8; **Figure 1D**) or flagellin (TLR5; **Figure 1E**) in the presence of IFN*γ* or with a cytokine maturation cocktail containing IL-1β, TNF-α; and PGE_2_ (**Figure 1B**, TNF-α not measured as this was added with stimulation cocktail). After stimulation, moDCs produced pro-inflammatory cytokines TNF-α, IL-12, and IL-6. In the presence of platelets however, pro-inflammatory cytokine production reduced significantly after all stimulations with an average residual cytokine production of 18.9%, 19.7%, and 43.4% for TNFα, IL-12p40, and IL-6 respectively after MPLA/IFN*γ* stimulation. Platelet-mediated inhibition of cytokine production was not observed for IL-6 production after R848/IFN*γ* stimulation and IL-12p40 production upon flagellin/IFN*γ* or cytokine cocktail stimulation, which both failed to induce a strong IL-12p40 response. The decrease in cytokine production was dependent on the number of platelets present (**Supplementary Figure S1A**). Additionally, in presence of platelets anti-inflammatory IL-10 production was induced for R848/IFN*γ* and IL-1β/TNFα/PGE_2_ stimulated moDCs (**Figures 1B, D**). MoDCs stimulated with MPLA/IFN*γ* and PAM3CSK/IFN*γ* display a non-significant trend of IL-10 induction in the presence of platelets (**Figures 1A, C**), whereas IL-10 production was not detectable for moDCs stimulated with Flagellin/IFN*γ* (**Figure 1E**). When immature moDCs were cultured with platelets without additional maturation stimulus, some donors showed an increase in cytokine production. This non-significant trend was most clear for IL-6 and IL-10 production by immature moDCs in presence of platelets (**Supplementary Figure 1B**).

Stimulation with MPLA/IFN*γ* or IL-1β/TNFα/PGE_2_ induced the highest expression of co-stimulatory molecules as measured by flow cytometry. Interestingly, platelets minimally affected the phenotype of MPLA/IFN*γ* or IL-1β/TNFα/PGE_2_ stimulated DCs, with only limited changes in expression of CD83 or CD40 and CD86, respectively (**Supplementary Figure S1C**; for gating strategy see **Supplementary Figure S2A**). In summary, platelets reduced moDC pro-inflammatory cytokine production after various DAMP/PAMP stimulations, despite minimal effects on co-stimulatory molecule expression.

### The Effect of Platelets on moDC Cytokine Production Is Mediated by a Soluble Factor

**Figure 2:**
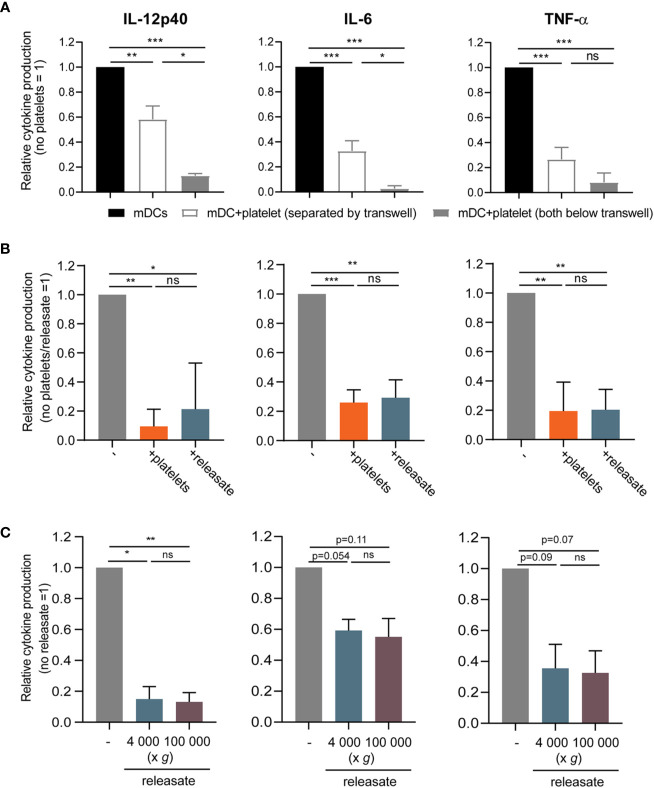
The effect of platelets on monocyte-derived dendritic cells (moDC) cytokine production is mediated by a soluble factor. **(A)** moDCs were stimulated with monophosporyl lipid A (MPLA)/IFN*γ* in a transwell system (pore size ≤ 0.3 µm) in absence or presence of thrombin receptor-activating peptide (TRAP)-activated platelets (platelet:DC ratio 40:1) that were either below the transwell (i.e. in the same compartment as the DCs) or separated by the transwell (*n*=4 donors; four independent experiments) (no platelets: IL-12p40 211-110714 pg/ml mean 23,068 pg/ml; IL-6 927.0 -127446 pg/ml mean 34,503 pg/ml; TNFα 352-44501 pg/ml mean 14,471 pg/ml). **(B, C)** moDCs were stimulated with MPLA/IFN*γ*  **(B)** in absence or presence of TRAP-activated whole platelets or platelet releasate centrifuged at 4,000 × *g* for 10 min ((equivalent) platelet:DC ratio 50:1 (*n*=4 donors; two independent experiments)) (no platelets: IL-12p40 24568-495930 pg/ml mean 233,399 pg/ml; IL-6 35500-62984 pg/ml mean 51,077 pg/ml; TNFα 6670-42400 pg/ml mean 24,768 pg/ml) or **(C)** in absence or presence of platelet releasate (equivalent platelet:DC ratio 50:1) centrifuged at 4000 g for 10 min (microvesicles in suspension) or additionally at 100 000 g for 1 h (no microvesicles in suspension) (*n*=3 donors; three independent experiments) (no platelets: IL-12p40 3370-10800 pg/ml mean 6,220 pg/ml; IL-6 5230-34900 pg/ml mean 17,168 pg/ml; TNFα 7540-32400 pg/ml mean 15,427 pg/ml). **(A–C)** After 48 h, supernatant was harvested and production of TNF-α, IL-12p40, and IL-6 was determined by ELISA. Differences in relative cytokine production (mean ± SEM) were determined using repeated measures one-way ANOVA with Tukey post testing. *p ≤ 0.05, **p ≤ 0.01.

Next, the contact dependency of platelet mediated inhibition of DC cytokine production was investigated. When platelets were cultured with DCs in a transwell system to prevent direct cell-cell contact, IL-12p40, IL-6, and TNF-α production by stimulated moDCs was also reduced although to a slightly lower extent, which possibly can be explained by a concentration gradient-induced effect of the transwell itself (**Figure 2A**). Hence platelets inhibited pro-inflammatory cytokine production by moDCs also without direct cell-cell contact.

To further explore the involvement of platelet-derived soluble mediators, the releasate secreted by activated platelets was tested. For this, isolated platelets were activated with the PAR1 agonist thrombin receptor activator peptide-6 (TRAP-6). After activation, the platelet suspension was centrifuged twice to remove whole platelets and cell fragments. Whole platelets and platelet releasate from the same platelet donor induced a comparable decrease of pro-inflammatory cytokine production by MPLA/IFN*γ* stimulated moDCs (**Figure 2B**), while TRAP-6 itself did not affect moDC viability or cytokine production (**Supplementary Figures S2B, C**). Lastly, to exclude involvement of microvesicles, the releasate was ultracentrifuged at 100,000 *g*. Releasate with or without ultracentrifugation equally inhibited pro-inflammatory cytokine production by stimulated moDCs (**Figure 2C**). Overall, platelets inhibit MPLA/IFN*γ*-stimulated moDC pro-inflammatory cytokine production *via* a soluble factor, and not through release of microvesicles.

As platelets are a major source of DC-tolerizing active TGF-β in plasma (46–50) and platelet-derived TGF-β was shown to inhibit the anti-tumor T cell response in a murine model (29, 51), we investigated the involvement of TGF-β in platelet-mediated DC inhibition. Both platelets and DCs possess machinery to activate latent TGF-β and release the mature 25-kDa dimer (29, 48). Upon chemical activation, inducing dissociation of the mature TGF-β dimer from latent TGF-β binding proteins (LTBPs), we could detect active TGF-β1 in platelet releasate (**Supplementary Figure S3A**), indicating that latent TGF-β is present and may potentially be activated in our culture system. However, blocking TGF-β in platelet releasate could not restore cytokine production by moDCs (**Supplementary Figure S3B-C**), indicating that TGF-β is not the responsible factor in the releasate for the inhibition of cytokine production by moDC.

### Platelet Releasate Affects Myeloid Conventional DC2 TNFα Production

In human blood, two types of myeloid or conventional DCs (myeloid cDC1 (CD141+) and cDC2 (CD1c+)) can be distinguished (32) and there is a subset of slan+ non-classical monocytes that show DC-like characteristics (52). To investigate to what extent our findings translate to human primary blood DCs, we isolated human myeloid CD1c+ conventional DC2 and slan+ non-classical monocytes from blood (**Supplementary Figure S4**).

**Figure 3:**
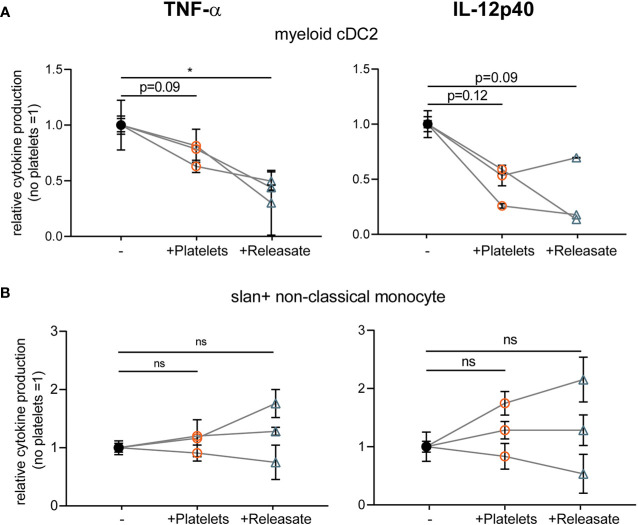
Platelet releasate affects myeloid conventional DC2 TNFα production. Primary human blood **(A)** myeloid CD1c+ conventional DC2 and **(B)** slan+ non-classical monocytes isolated from buffy coats were **(A, B)** stimulated with R848 in absence or presence of platelets or platelet releasate (centrifuged 4000 × *g* 10’) ((equivalent) platelet: dendritic cells DC ratio 50:1). After 24 h, supernatant was harvested and production of TNF-α (left; no platelets myeloid cDC2 141-364 pg/ml mean 284.7 pg/ml; slanDC 959-10100 pg/ml mean 4,306.5 pg/ml) and IL-12p40 (right; no platelets myeloid cDC2 111-335 pg/ml mean 226 pg/ml; slanDC 930-1530 pg/ml mean 1,238.3 pg/ml) was determined by ELISA (*n*=3 donors; three independent experiments). Differences in relative cytokine production (mean ± SEM) were determined using repeated measures one-way ANOVA with Tukey post testing. *p ≤ 0.05.

After overnight rest, isolated myeloid cDC2’s and slan+ non-classical monocytes were stimulated with R848 as primary DCs were found to not respond to stimulation with MPLA/IFN*γ*  (data not shown; (45)). Interestingly, platelet releasate significantly decreased TNF-α production by myeloid cDC2 and a trend for IL-12p40 inhibition was observed (**Figure 3A**), whereas cytokine production by slan+ non-classical monocytes was not affected (**Figure 3B**). Unfortunately, IL-10 production could not be detected for any of the primary DC subsets tested (*data not shown*). These findings confirm that the platelet-mediated effects found on moDC cytokine production could partly be validated in a subset of primary human blood DCs, namely myeloid cDC2s.

### Platelets and Platelet Releasate Inhibit T Cell Activation and Skewing of Naïve T Cells Toward an IFNγ+ Th1 Phenotype by moDCs

**Figure 4:**
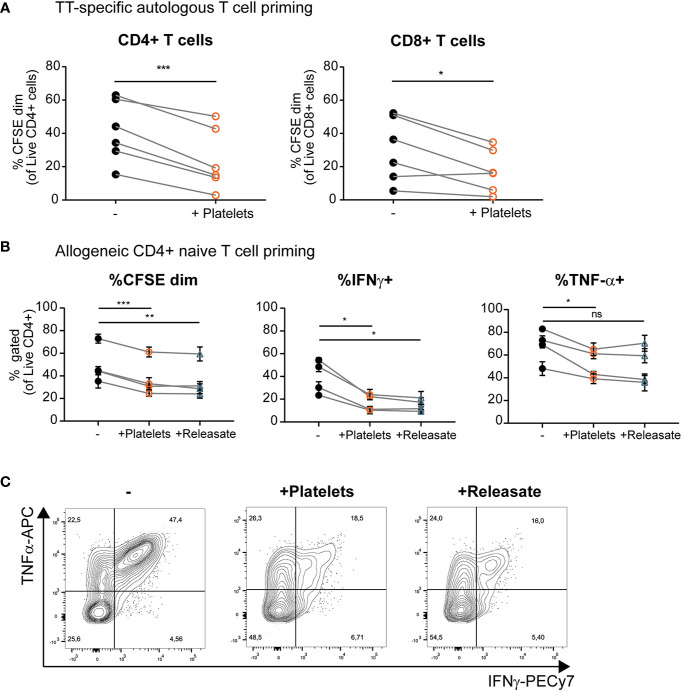
Platelets and platelet releasate inhibit T cell activation and skewing of naïve T cells toward an IFN*γ*+ Th1 phenotype by monocyte-derived dendritic cells (moDCs). **(A)** After 1 h of pre-incubation with tetanus toxoid (TT), moDCs were stimulated with monophosporyl lipid A (MPLA)/IFN*γ* in absence or presence of whole platelets (platelet: dendritic cells DC ratio 40:1). After 48 h, DCs were harvested and incubated 1:10 with CFSE-labeled autologous lymphocytes. After 8 days, T cell proliferation was determined using flow cytometry (*n*=6 donors; six independent experiments) **(B, C)** moDCs were stimulated with MPLA/IFN*γ* in absence or presence of whole platelets or platelet releasate (centrifuged 4000 x *g* 10’)((equivalent) platelet:DC ratio 50:1). After 48 h, DCs were harvested and incubated 1:10 with CFSE labeled allogeneic naïve CD4+ CD45RA+ T cells. After 10 days, T cell proliferation and intracellular cytokine production was determined by flow cytometry (*n*=4 donors; two independent experiments). **(C)** Representative plots of intracellular TNFα and IFN*γ* production by naïve CD4+ CD45RA+ T cells stimulated as described above. **(A)** Differences in proliferation were determined using paired t-tests. **(B)** Differences in proliferation and cytokine production (mean ± SEM) were determined by repeated measures one-way ANOVA with Tukey post testing. *p ≤ 0.05, **p ≤ 0.01, ***p ≤ 0.001.

Above we have shown that platelets and platelet releasate affect pro-inflammatory cytokine production by DCs, but the effect on subsequent T cell responses remains largely unresolved. To investigate the effect of platelets on DC-mediated T cell stimulation, moDCs were loaded with tetanus toxoid (TT) and subsequently stimulated with MPLA/IFN*γ* in presence of platelets. MoDCs stimulated in presence of platelets induced less TT-specific autologous CD4+ and CD8+ T cell proliferation (**Figure 4A** and **Supplementary Figure S5A**). A similar decrease in the ability of moDCs to induce allogeneic CD4+ but not CD8+ proliferation under influence of platelets or platelet releasate was found (**Figure 4B** and **Supplementary Figures S5B, C**).

Another interesting aspect of DC biology is their ability to skew naïve T cells toward a specific T helper phenotype upon the initiation of an immune response. MoDCs were cultured with MPLA/IFN*γ* in presence of whole platelets and platelet releasate. In a subsequent co-culture with naïve CD4+ CD45RA+ T cells, moDCs stimulated in presence of platelets or platelet releasate induced less allogeneic proliferation of naïve T cells (**Figure 4B**, left panel, gating strategy in **Supplementary Figure S6A**). Additionally, divided T cells (CFSEdim) displayed decreased IFN*γ* production compared to control co-cultures where moDCs were stimulated in the absence of platelets or platelet releasate (**Figures 4B**, middle panel, and **4C**). Furthermore, the divided T cells produced less TNFα compared to control co-cultures where moDCs were stimulated in the absence of platelets but not platelet releasate (**Figures 4B**, right panel, and **4C**). No significant production of IL-10 was detected in any of the DC-T cell co-cultures with or without platelet releasate (*data not shown*).

To determine whether effects on T cell skewing are dependent on the type of stimulation, cultures were performed with moDCs stimulated with TNFα, IL-1β; and PGE_2_. We observed an increase in moDC anti-inflammatory IL-10 production when stimulated in presence of platelet releasate (**Supplementary Figure S6B**). Simultaneously, we observed a decrease in IL-6 production (**Supplementary Figure S6B**; TNFα could not be analyzed as it was present in the stimulation cocktail) and also a decreased ability to induce naïve T cell proliferation as measured by CFSE dilution by flow cytometry (**Supplementary Figure S6C**, left panel). No effect was found on the IFN*γ* production of these naïve T cells compared to controls (**Supplementary Figure S6C**, right panel). All in all, platelets and platelet releasate can decrease the ability of moDCs to induce both memory and naïve T cell proliferation. Additionally, the ability of MPLA/IFN*γ* stimulated moDCs to induce IFN*γ*+ Th1 responses was reduced when stimulated in presence of platelets or platelet releasate.

## Discussion

The mechanisms underlying TRIM are hard to investigate, but the role platelets themselves may play in this immune modulatory phenomenon after transfusion is increasingly being acknowledged (12, 31). Mechanisms of immune suppression as a result of TRIM are worth elucidating as these might be both beneficial (e.g. decreased graft rejection) or detrimental (e.g. recurrence resected malignancies) for the patient (14, 15, 18). However, much is still unclear about platelet-mediated immune modulation on DCs and whether platelets also affect subsequent ability of DCs to prime naïve and re-activate memory T cells. Here we show that a soluble factor secreted by platelets affects both moDC and primary myeloid CD1c+ conventional DC2 cytokine production under different stimulation conditions. Furthermore, these platelet-modulated moDCs are less capable to induce T cell proliferation and to skew naïve T cells toward a Th1 IFN*γ* ^+^ phenotype.

Activation of transfused platelets could be induced by the so-called platelet storage lesion (53) or after transfusion by vascular damage in the recipient, which is frequently observed in hemato-oncological patients (7). Our observation of activated platelets decreasing pro-inflammatory responses at a potential physiological site of tissue damage makes sense when considering that here platelet-mediated immune suppression could favor wound healing over an excessive inflammatory response. In the setting of TRIM however, interaction of activated platelets with DCs might also occur in absence of tissue injury. This is an important mechanism to consider especially in thrombopenic and immune-compromised hemato-oncological patients whom are the primary recipients of platelet transfusions (7). Within the vasculature, transfused activated platelets might come across blood DCs (53–55) or encounter APCs while passing through the white and red pulp of the spleen (56).

Previous studies have reported contrasting results on the effect of platelets on DC activation. Platelets have generally been found to increase activation of DCs in absence of an additional stimulus (36, 39–42), while in presence of TLR-4 stimulus (i.e. LPS) platelets suppress DCs pro-inflammatory capacity or even induce an anti-inflammatory phenotype (33, 34, 37, 38, 43). In line, here platelets are shown to decrease pro-inflammatory cytokine production by DCs after stimulation with various TLR-dependent as well as TLR-independent agonists, implicating a more generic effect. Hagihara and colleagues described previously that moDCs activated with LPS in presence of platelets showed increased IL-10 production (33). Here, in agreement with a general immune suppressive effect, we found an increased IL-10 production for R848/IFN*γ*- and IL-1β/TNFα/PGE_2_- stimulated moDCs in the presence of platelets.

Despite the profound effect of platelet-derived mediators on moDC cytokine production, the effect on costimulatory marker expression was limited. Previous studies have shown varying effects of platelets on co-stimulatory marker expression by DCs (33–36, 38, 43). We have previously observed that inhibition of cytokine production by moDCs is not always linked to reduced co-stimulatory marker expression (57). To gain more insight in the mechanism behind reduced cytokine expression with limited effects on co-stimulatory marker expression, it is important to investigate at which level of the DC activation signaling cascade platelets or platelet-derived factors interfere. Irrespectively, we have shown that moDCs stimulated in presence of platelets showed decreased ability to activate a TT-specific memory response as well as induce proliferation and Th1 IFN*γ*+ skewing of naïve T cells in an allogeneic setting.

A number of studies have also reported on direct effects of platelets on T cell proliferation and cytokine production (29, 51, 58). Despite extensive washing, complex formation between platelets and DCs might occur during DC stimulation (13, 41). However, our experiments using platelet releasate where platelet-derived factors were washed away before co-culture of DCs and T cells show very comparable effects on T cell proliferation and cytokine production. This demonstrates that the effects of platelets on T cell proliferation and cytokine production are highly likely to be mediated through a direct effect of platelets on DC effector function.

Interestingly, experiments performed with platelet releasate showed that the platelet-mediated effects on DC cytokine production and T cell priming capacity are primarily mediated by soluble factors and are not mediated by microvesicles. We are the first to show that the platelet-mediated effects on DC cytokine production are not mediated by microvesicles. Microvesicles were previously shown to affect HLA II and CD80 expression as well as phagocytic capacity of immature moDCs differentiated in the presence of microparticles shed by stored platelets (38), and were shown to inhibit human plasmacytoid DC pro-inflammatory cytokine production (59).

The immune modulatory factor(s) secreted by platelets that affect DC function remain(s) to be identified. Platelets are capable of secreting a wide range of mediators that could influence DC biology (12, 30). For example, TGF-β is activated and expressed by platelets and is known to alter DC effector functions by directing toward a tolerance-inducing phenotype (46–49). Here we show for the first time that platelet-derived TGF-β is unlikely to be involved in platelet-induced inhibition of DC. Next to TGF-β, platelets are able to express and secrete (soluble) CD40L, but as this molecule usually induces DC maturation and upregulation of cytokine production (40, 60, 61) (s)CD40L is an unlikely mediator of the results described here. For CXCL4 (platelet factor 4) contrasting literature exists on its effects on DC function, with reports documenting anti-inflammatory effects (62) as well as potentiated response to TLR ligands upon culture of moDCs in presence of CXCL4 (63, 64). In addition, multiple factors could act together to mediate suppression of pro-inflammatory cytokine production by moDC.

Further research is warranted to elucidate which platelet-derived factors affect DC activation. We propose for upcoming studies to first validate whether the platelet-derived factor is (partly) of a proteinaceous nature, or whether e.g. platelet metabolites are responsible for the observed effects. An unbiased approach using liquid chromatography, potentially in combination with mass spectrometry (as described in (29)) would be interesting to identify the responsible platelet-derived factor(s) involved in the inhibition of DC effector functions.

Platelet-mediated inhibition was not only found for moDC but also observed for myeloid conventional DC2’s isolated from the blood, in line with previous research using platelet concentrates (36, 37). However, as these studies were performed in a whole blood culture, indirect effects on DCs *via* other cells could not be excluded. Here we show that platelet-derived soluble mediators inhibit pro-inflammatory cytokine production of purified myeloid cDC2, thus proving a direct effect of platelet (releasate) on primary DCs. As myeloid cDC2s have an extensive array of PRRs and a widespread tissue distribution, platelet-mediated inhibition could affect immunity against a wide range of pathogens (32). Interestingly, cytokine production of slan+ non-classical monocytes was not affected, which may be explained by their intrinsic capacity of higher production of IL-12 and TNFα compared to myeloid cDCs (65, 66).

In summary, we have shown that platelets reduce pro-inflammatory cytokine production of both moDCs and primary myeloid CD1c+ conventional DC2s. This inhibition is mediated by a soluble factor, and was observed in the presence of a range of both TLR-dependent as well as TLR-independent stimuli. Moreover, moDCs stimulated in the presence of platelets show a decreased ability to induce (antigen-specific) T cell proliferation as well as naïve T cell skewing toward a Th1 IFN*γ* ^+^ phenotype. Together, these data show that platelets can influence the adaptive T cell response by inhibition of DC activation, with potential implications in platelet transfusions.

## Data Availability Statement

The raw data supporting the conclusions of this article will be made available by the authors, without undue reservation.

## Ethics Statement

The studies involving human participants were reviewed and approved by the Ethical Advisory Council of Sanquin Blood Supply Foundation and are in accordance with the declaration of Helsinki and Dutch regulations. The patients/participants provided their written informed consent to participate in this study.

## Author Contributions 

AS and JS conceptualized and designed the study, acquired and interpreted data, wrote and revised the manuscript. DS and GS acquired data. JZ, SH, and AB conceptualized and designed the study, interpreted data and critically revised the manuscript. All authors contributed to the article and approved the submitted version.

## Funding

This work was supported by Sanquin Blood Supply Foundation (grant PPOC 17-44).

## Conflict of Interest

The authors declare that the research was conducted in the absence of any commercial or financial relationships that could be construed as a potential conflict of interest.

## Abbreviations

CFSE, Carboxyfluoresceïne succinimidyl ester; DAMP, damage-associated molecular pattern; FNHTR, febrile non-hemolytic transfusion reactions; IFNγ; interferon γ; IL-1β; interleukin 1β; LTBP, latent TGF-β binding protein; MPLA, monophosphoryl lipid A; PAM3CSK4, pam3CysSerLys4; PAMP, pathogen-associated molecular pattern; PGE_2_, prostaglandin E2; PMA, phorbol myristate acetate; TGFβ; transforming growth factor β; TNFα; tumor necrosis factor α; TRALI, transfusion related acute lung injury; TRAP-6, thrombin receptor-activating peptide 6; TRIM, transfusion related immune modulation; TT, tetanus toxoid.
