# Supplementary material for: Inhibition of Dendritic Cell Activation and Modulation of T Cell Polarization by the Platelet Secretome
Source: Front Immunol. 2021 Feb 25;12:631285. doi: 10.3389/fimmu.2021.631285 (PMC7961920; doi:10.3389/fimmu.2021.631285)
Supplement: Supplementary file 1 [file DataSheet_1.pdf]

Supplementary Figures

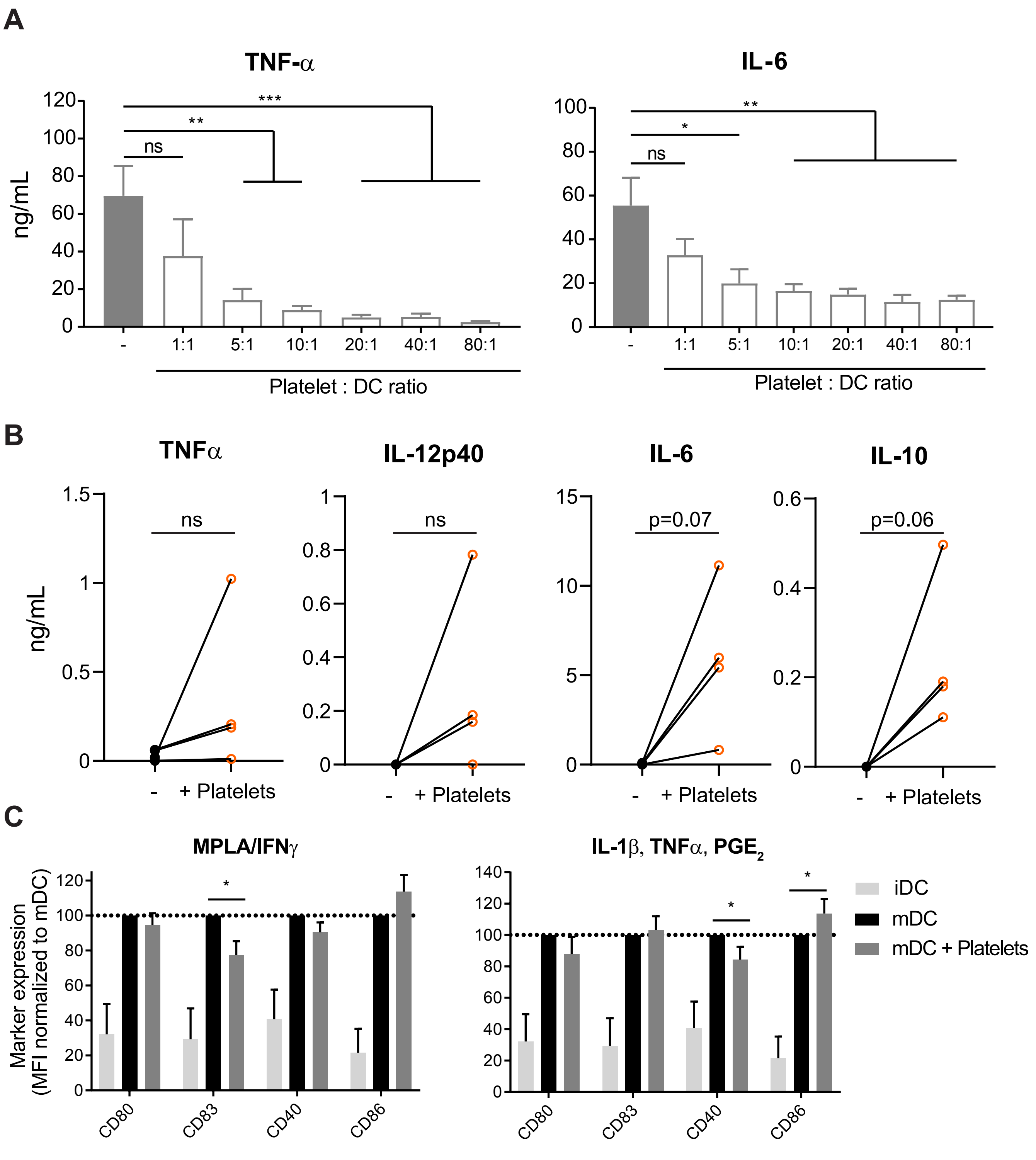

Supplementary Figure S1 – Titration of platelet numbers and effect of platelets on moDC co-stimulatory marker expression (A) moDCs were stimulated with MPLA/IFNγ in the absence or presence of increasing numbers of platelets (*n*=3 donors; 3 independent experiments). After 48h, supernatant was harvested and production of TNF-α and IL-6 measured by ELISA. Differences in cytokine production (mean±SD) were determined using repeated measures one-way ANOVA with Bonferroni post testing. **(B)** moDCs were incubated for 24h with freshly isolated platelets (platelet:DC ratio 40:1) without addtional maturation stimuli. After 24h, supernatant was harvested and production of TNFα, IL-12p40, IL-6 and IL-10 was measured by ELISA. Differences in cytokine production were determined by paired t-test (*n*=4 donors; 4 independent experiments). **(C)** moDCs were left untreated or stimulated with MPLA/IFNγ (*n*=9;donors; 9 independent experiments) or IL-1β/TNFα/PGE_2 (*n*=3; 2 independent experiments) in absence or presence of platelets (platelet:DC ratio 40:1). After 48h, DCs were harvested and expression of costimulatory markers was measured by flow cytometry. Differences in normalized MFI (mean±SD) were determined by paired t-test.

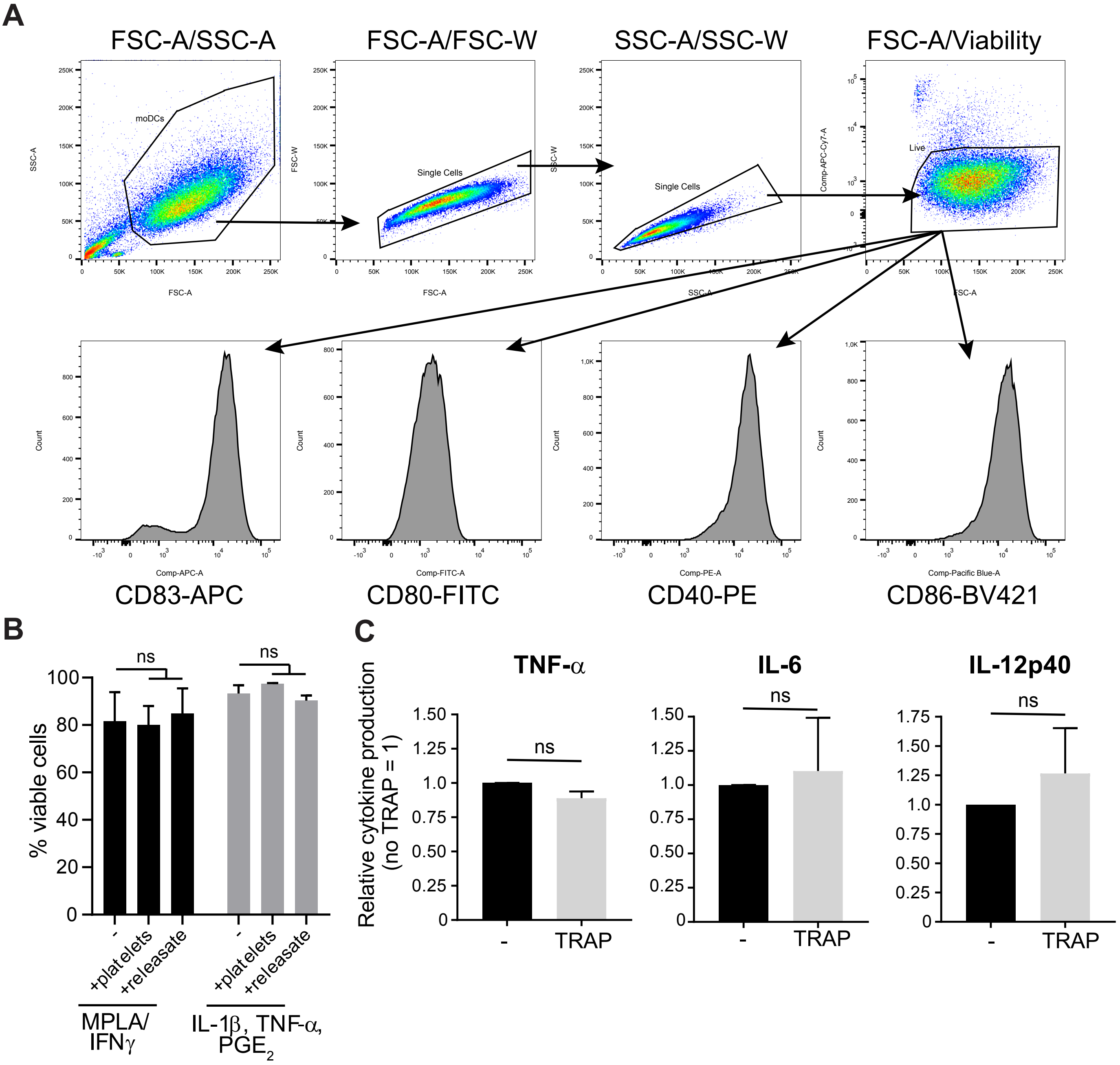

Supplementary Figure S2 – Gating strategy moDCs and effects of platelets or TRAP on moDC viability and cytokine production (A) representative gating strategy mature moDCs (here matured using IL-1β/TNFα/PGE$_2$). moDCs were gated based on size and granularity (FSC-A/SSC-A), two gates to remove duplicates (FSC-A/FSC-W & SSC-A/SSC-W) and viability (FSC-A/Viability dye) and subsequently MFI of CD83-APC, CD80-FITC, CD40-PE and CD86-BV421 was determined. **(B)** moDCs were left untreated or stimulated with MPLA/IFNγ or IL-1β/TNFα/PGE$_2$(n=3 donors; 2 independent experiments) in absence or presence of platelets or platelet releasate (centrifuged 4000 x g 10') ((equivalent) platelet:DC ratio 40:1). After overnight incubation, DCs were harvested and viability was measured by flow cytometry using near-IR fixable viability dye. Differences in viability (mean±SD) were determined using repeated measures one-way ANOVA using Bonferroni post-testing **(C)** moDCs were stimulated with MPLA/IFNγ in the absence or presence of equivalent amount of TRAP-6 representative of 40:1 platelet:DC co-culture. After 48h, supernatant was harvested and cytokine production was determined by ELISA (n=4 donors; 4 independent experiments). Differences in cytokine production (mean±SD) were determined by paired t-test.

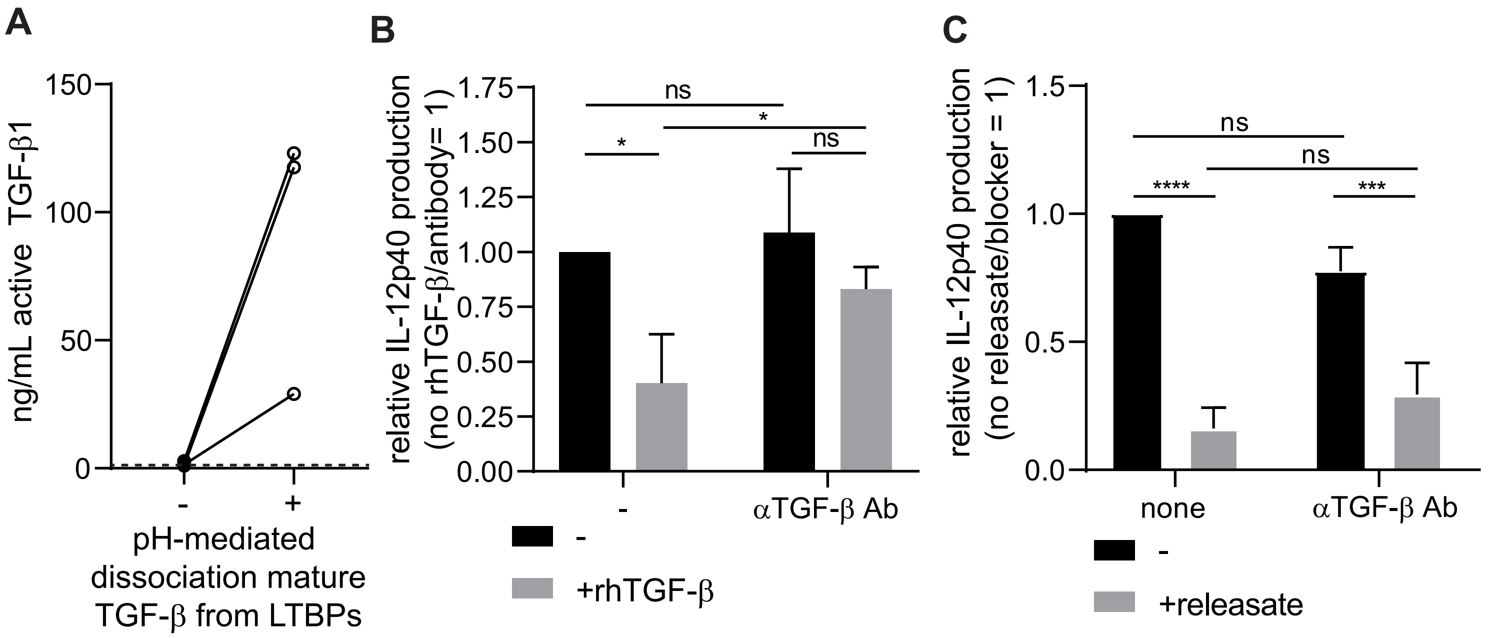

Supplementary Figure S3 – Blocking TGF-β does not rescue moDC cytokine production in presence of platelet releasate **(A)** measurement of active TGF-β1 in platelet releasate (*n*=3 releasate batches composed of platelets from 4-5 donors; 3 independent experiments) by ELISA with and without sample activation. pH-mediated sample activation induces dissociation of mature TGF-β from LTBPs, which is required before TGF-β can exert biological activitiy. Dashed line represents level of active TGF-β1 in culture medium after sample activation. **(B-C)** moDCs were stimulated with MPLA/IFNγ in the absence or presence of **(B)** 5ng/mL rhTGF-β to test efficacy of αTGF-β blocking antibody or **(C)** platelet releasate (centrifuged 4000 x g 10') (equivalent DC:platelet ratio 1:50) in the presence or absence of an anti-TGF-β mAb (Clone 1D11). After overnight incubation, supernatant was harvested and production of IL-12p40 determined by ELISA (*n*=3 donors; 1 experiment). Differences in cytokine production (mean±SD) were determined using two-way ANOVA with Tukey's test for multiple comparisons.

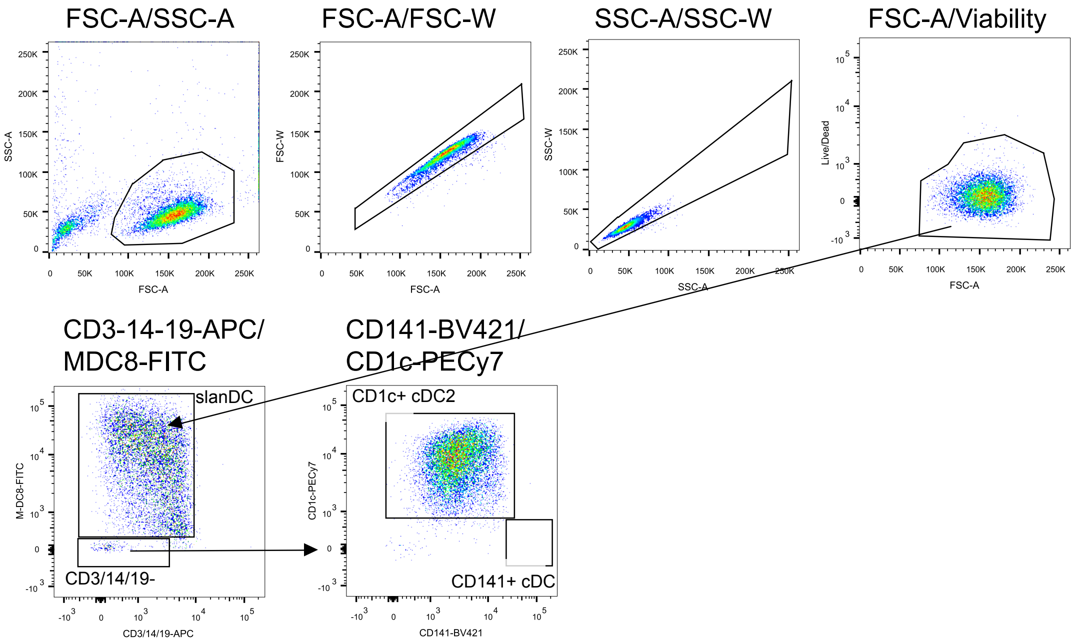

Supplementary Figure S4 – Gating strategy for isolation of primary DCs from buffy coat Primary blood DCs were sorted from ELUTRA enriched buffy coat derived PBMC fraction. Cells were gated based on FSC/SSC, two gates to remove duplicates and live cells. Hereafter, slan+ non-classical monocytes were gated as CD3/14/19^{-} and M-DC8^{+}. Myeloid conventional DC2 were gated from CD3/14/19^{-} as CD1c^{+}. Also myeloid conventional DC1 were gated as CD3/14/19^{-} CD141^{+} but cell numbers were too low for continuation in experiments. Representative figure from one of three independent experiments.

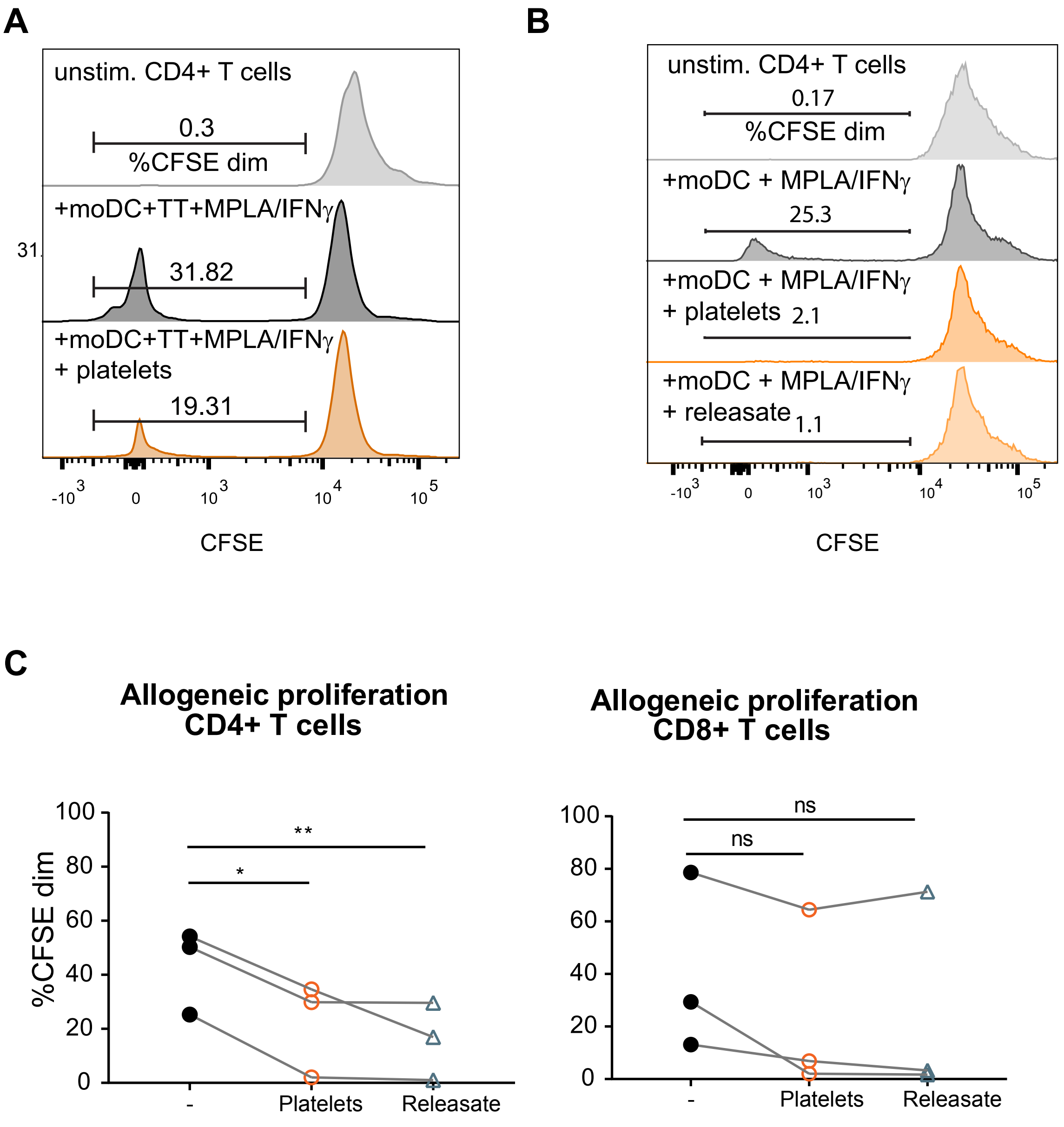

Supplementary Figure S5 – Autologous and allogeneic T cell proliferation in the presence of platelets and platelet releasate (A-B) Example overlay histograms of CFSE signal of **(A)** TT specific autologous T cell proliferation and **(B)** allogeneic CD4+ T cell proliferation **(A-C)** When indicated, moDCs were loaded with TT after which moDCs were stimulated with MPLA/IFNγ in absence or presence of whole platelets or **(B-C)** platelet releasate (centrifuged 4000 x g 10') ((equivalent) platelet:DC ratio 40:1). **(A-C)** After 48 hours, DCs were harvested and incubated 1:10 with CFSE labelled **(A)** autologous or **(B-C)** allogeneic total lymphocytes. After 8 days, T cell proliferation was determined using flow cytometry (*n*=3 donors; 3 independent experiments). **(C)** Differences in proliferation (mean±SD) were determined using one-way ANOVA with Bonferroni post testing.

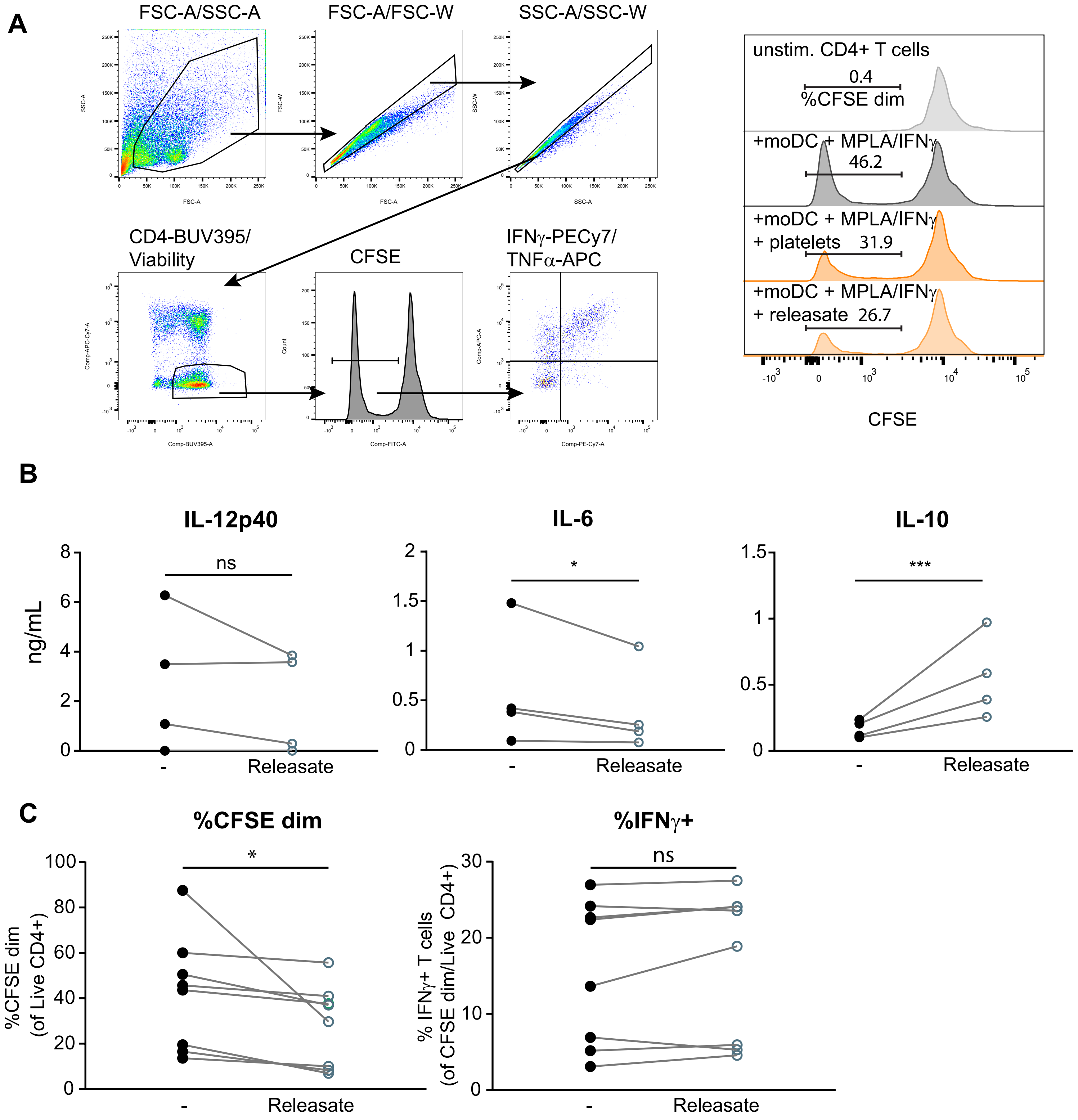

Supplementary Figure S6 – Effect of platelet releasate on gold-cocktail stimulated moDC effector functions **(A)** representative plots of gating strategy naive T cell cultures. T cells were gated based on FSC-A/SSC-A, two gates to remove duplicates (FSC-A/FSC-W and SSC-A/SSC-W), viability and CD4+, after which the CFSE dim fraction was used to determine proliferation (detail in right overlay panel) and proliferated cells were gated for evaluation of TNFα and IFNγ production **(B-C)** moDCs were stimulated with cytokine cocktail containing TNF-α, IL-1β and PGE_2 in absence or presence of platelet releasate (centrifuged 4000 x g 10') (equivalent platelet:DC ratio 50:1). **(B)** After 48 hours, supernatant was harvested and production of IL-12p40, IL-6 and IL-10 determined by ELISA (*n*=4 donors; 2 independent experiments). **(C)** After 48 hours, DCs were harvested and cultured in 1:10 ratio with CFSE labelled naïve CD4+ CD45RA+ allogeneic T cells. After 10 days, T cell proliferation and intracellular IFNγ production was determined by flow cytometry (*n*=7 donors; 4 independent experiments). **(B-C)** Differences in DC cytokine production as well as differences in T cell proliferation and cytokine production (mean±SD) were determined by paired t-tests.
